# A comparison of US and Australian men’s values and preferences for PSA screening

**DOI:** 10.1186/1472-6963-13-388

**Published:** 2013-10-05

**Authors:** Kirsten Howard, Alison T Brenner, Carmen Lewis, Stacey Sheridan, Trisha Crutchfield, Sarah Hawley, Matthew E Nielsen, Michael P Pignone

**Affiliations:** 1 Sydney School of Public Health, University of Sydney, Edward Ford Bldg (A27), Sydney, NSW 2006, Australia; 2 School of Public Health, University of Washington, Seattle, WA, USA; 3 Department of Medicine, University of North Carolina, Chapel Hill, NC, USA; 4 Sheps Center for Health Services Research, University of North Carolina, Chapel Hill, NC, USA; 5 Lineberger Comprehensive Cancer Center, University of North Carolina, Chapel Hill, NC, USA; 6 Department of Internal Medicine, University of Michigan, Ann Arbor, MI, USA; 7 Ann Arbor VA Health System, Ann Arbor, MI, USA; 8 Department of Urology, University of North Carolina, Chapel Hill, NC, USA

## Abstract

**Background:**

Patient preferences derived from an assessment of values can help inform the design of screening programs, but how best to do so, and whether such preferences differ cross-nationally, has not been well-examined. The objective of this study was to compare the values and preferences of Australian and US men for PSA (prostate specific antigen) screening.

**Methods:**

We used an internet based survey of men aged 50–75 with no personal or family history of prostate cancer recruited from on-line panels of a survey research organization in the US and Australia. Participants viewed information on prostate cancer and prostate cancer screening with PSA testing then completed a values clarification task that included information on 4 key attributes: chance of 1) being diagnosed with prostate cancer, 2) dying from prostate cancer, 3) requiring a biopsy as a result of screening, and 4) developing impotence or incontinence as a result of screening. The outcome measures were self reported most important attribute, unlabelled screening test choice, and labelled screening intent, assessed on post-task questionnaires.

**Results:**

We enrolled 911 participants (US:456; AU:455), mean age was 59.7; 88.0% were white; 36.4% had completed at least a Bachelors’ degree; 42.0% reported a PSA test in the past 12 months. Australian men were more likely to be white and to have had recent screening. For both US and Australian men, the most important attribute was the chance of dying from prostate cancer. Unlabelled post-task preference for the PSA screening-like option was greater for Australian (39.1%) compared to US (26.3%) participants (adjusted OR 1.68 (1.28-2.22)). Labelled intent for screening was high for both countries: US:73.7%, AUS:78.0% (p = 0.308).

**Conclusions:**

There was high intent for PSA screening in both US and Australian men; fewer men in each country chose the PSA-like option on the unlabelled question. Australian men were somewhat more likely to prefer PSA screening. Men in both countries did not view the increased risk of diagnosis as a negative aspect, suggesting more work needs to be done on communicating the concept of overdiagnosis to men facing a PSA screening decision.

**Trial registration:**

This trial was registered at ClinicalTrials.gov (NCT01558583).

## Background

Whether to undergo prostate-specific antigen (PSA) screening is a difficult decision for middle-aged men. Prostate cancer is common, and causes over 29000 deaths per year in the US and approximately 3000 per year in Australia [1,2]. However, PSA screening, at best, seems to produce only a small reduction in prostate cancer mortality and has considerable downsides [3,4]. These downsides include increases in the number of prostate biopsies (which can be painful and have a risk of causing infection), as a result of abnormal PSA screen results; overdiagnosis, (i.e. the detection of cancers that would never become clinically apparent or problematic); and increased treatment and treatment-related adverse effects (impotence and incontinence) [3-5].

Because the number of men who benefit from screening is small and the downsides common, guideline-making organisations often recommend a shared decision making approach incorporating an individual’s own values and preferences: “men thinking about prostate cancer screening should make informed decisions based on available information, discussion with their doctor, and their own views on the benefits and side effects of screening and treatment” [6] (American Cancer Society); “…whether or not to be tested for prostate cancer is a matter of individual choice” [7] (Cancer Council of Australia).

Despite these recommendations, surveys suggest that few men are adequately informed about the benefits and downsides of screening [8,9] and that testing rates are high in many western countries, including the US and Australia [10-12].

The objective of our study was to compare how Australian and US men value different attributes of PSA screening and whether such values affect their preferences for whether to be tested or not.

## Methods

### Overview

We surveyed male members of on-line panels in the US and Australia. Details of methods have been previously reported elsewhere [13]; a brief summary of methods is provided below. This paper focuses on cross country comparisons of values and preferences. The results of the comparison of different values clarification methods (VCM) have been previously published [13].

### Participant eligibility and recruitment

We used the online panels maintained by an international research firm Survey Sampling International (SSI) to recruit a target of 900 men (450 US, 450 Australia). Participants aged 50–75 who had no personal or family history of prostate cancer were targeted. Prior testing history was assessed but not used to determine eligibility. Those with visual limitations or inability to understand English were excluded.

### Study flow

The entire study was performed online. After eligibility was determined and consent obtained, participants received basic information about prostate cancer and PSA screening, completed demographic questions, and were then randomized by SSI on a 1:1:1 basis, stratified by country, to one of three values clarification methods (VCM): 1) an implicit values clarification method (a balance sheet of key test attributes); 2) a rating and ranking task; or 3) a discrete choice experiment (DCE), followed by post-task questions.

### Selection of attributes and levels

For all values clarification methods, we described the PSA screening decision (whether or not to be screened) in terms of four key attributes: 1) chance of being diagnosed with prostate cancer, 2) chance of dying from prostate cancer, 3) chance of requiring a biopsy as a result of screening, and 4) chance of developing impotence or incontinence as a result of screening. The attributes and the range of levels of the attributes included were drawn from the literature and our own previous work [3,5].

### Study outcomes

**Figure 1:**
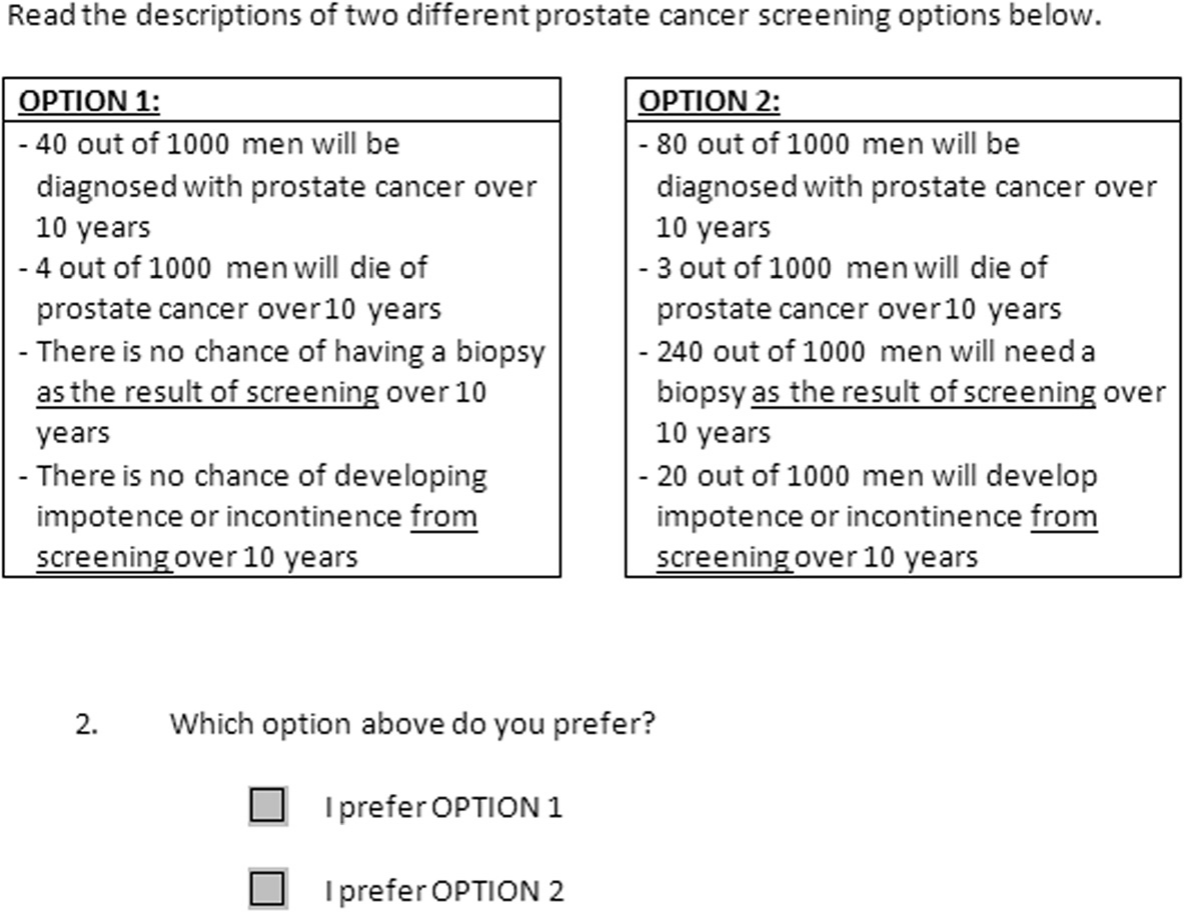
Unlabelled test preference question.

Our three main outcomes of interest were 1) the participant-reported most important attribute (“Which ONE feature of prostate cancer screening is most important to you?” chosen from the four attributes above), 2) the post-task testing preference, based on a question that included two unlabelled options described in terms of the key decision attributes and designed to mimic screening or no screening options - we call this “unlabelled test preference” (Figure 1); and 3) a single post-task question about intent to be screened with PSA, based on a Likert scale (from strongly disagree to strongly agree, with agree and strongly agree considered as positive intent to be screened) - we refer to this as the “labelled test preference”.

### Analyses

We performed initial descriptive analyses of all variables with means and proportions. We used chi-square and ANOVA for bivariate analyses across the two country groups and calculated unadjusted odds ratios (OR) and 95% confidence intervals of OR. Because of baseline demographic differences between the US and Australian men, we also performed multivariate analyses using logistic regression, and adjusted for potential confounders, including age, race, education, income, and prior PSA testing.

We also assessed the relationship between the participant-reported most important attribute and unlabelled test preference. A priori we expected that if mortality benefit is the most important attribute, then unlabelled test preference should favour the screening-like test option, and more men choosing the screening-like option would also choose chance of death as the most important attribute. Similarly, if potential harms such as impotence/incontinence, or the chance of diagnosis were most important, then we might expect men to prefer the unlabelled test option that described no screening.

### Ethical considerations

This study was approved by the University of North Carolina - Chapel Hill Institutional Review Board on April 28, 2011 (Study number 11–0861) and is registered through ClinicalTrials.gov (NCT01558583).

## Results

We screened 2336 individuals from October, 12 – 27, 2011. Of these, 595 were ineligible, 705 declined participation before being randomised and 1036 were randomized. Of these 1036, 911 (87.9%) completed the full survey. Participant characteristics are shown in Table 1. We noted potentially important differences across country groups in the proportion of white participants, education level and the proportion reporting PSA testing within the past 12 months.

**Table 1:** characteristics of participants overall (n = 911) and by country

	**Overall**	**United States**	**Australia**	**p-value**
	**(n = 911)**	**(n = 456)**	**(n = 455)**	
**Mean age (SD)**	59.8 (5.6)	59.7(5.5)	59.8 (5.7)	p = 0.730
**Ethnicity**	88.0%	82.5%	93.6%	p < 0.0001
(% White)				
**Education**				p < 0.0001
Less than high school graduate	7.2%	2.0%	12.5%	
High school graduate or some college	56.3%	51.1%	61.5%	
College graduate	36.4%	46.9%	25.9%	
**Income**				p = 0.049
<$30,000	24.0%	23.9%	24.2%	
$30,000-59,999	30.4%	32.2%	28.6%	
> = $60,000	35.9%	36.8%	34.9%	
Prefer Not to Answer	9.7%	7.0%	12.3%	
**Employment**				p = 0.002
Employed	36.4%	36.8%	36.0%	
Retired	39.3%	37.1%	41.5%	
Other	24.3%	26.1%	22.5%	
**PSA testing**				p = 0.103
within past year	42.0%	38.6%	45.5%	
> 1 year ago	20.9%	21.7%	20.0%	
Never/Don’t know	37.1%	39.7%	34.5%	

### Main outcomes

The participant-reported most important attribute from the single post-task questionnaire indicated similar proportions of US and Australian respondents choosing specific test attributes as the most important (Table 2). Australian men were no more likely than US men to choose mortality as the most important attribute: 41.8% vs 39.7% (unadjusted OR 1.05 (0.89-1.23); adjusted OR 1.14 (0.88 - 1.49)). Australian men were slightly less likely to choose impotence/incontinence as the most important attribute (15.4% vs 21.3%, p = 0.022, unadjusted OR 0.67 (0.48 - 0.94)). This effect was slightly attenuated after adjustment for confounders (adjusted OR 0.72 (0.51 - 1.03)).

**Table 2:** Most important attribute from post-task questionnaire

	**US (n = 456)**	**AUS (n = 455)**	**p-value (pairwise)**
Chance of being diagnosed over 10 years	27.2%	32.3%	0.091
Chance of dying over 10 years	39.7%	41.8%	0.526
Chance of needing a biopsy from screening over 10 years	11.8%	10.5%	0.536
Chance of impotence/incontinence over 10 years	21.3%	15.4%	0.022

### Unlabelled test preference

In terms of unlabelled test preference, Australian men were significantly more likely (39.1%) to prefer the PSA-like option (as opposed to the no screening option), compared to US men (26.3%), p < 0.0001, unadjusted OR 1.46 (1.34 – 1.56). This difference remained after adjustment for potential confounders (adjusted OR 1.68 (1.28 – 2.22).

### Does the most important attribute influence unlabelled test preference?

We assessed the relationship between the most important attribute and unlabelled test preference. Overall, the relationship between most important attribute and unlabelled test preference was generally as expected: the proportion of men choosing the chance of dying as most important was lower for those choosing the no screening-like option, and the proportion of men choosing impotence/incontinence as most important was higher for those choosing the no screening-like option (Table 3).

**Table 3:** Relationship between most important attribute and unlabelled test preference

**Attribute**	**Overall (n = 911)**				**US (n = 456)**				**AUS (n = 455)**			
	**Unlabelled test preference**				**Unlabelled test preference**				**Unlabelled test preference**			
	**No screening like option (n = 613)**		**Screening-like option (n = 298)**		**No screening like option (n = 336)**		**Screening-like option (n = 120)**		**No screening-like option (n = 277)**		**Screening-like option (n = 178)**	
	**N**	**%**	**N**	**%**	**N**	**%**	**N**	**%**	**N**	**%**	**N**	**%**
Chance of being diagnosed over 10 years	172	28.1%	99	33.2%	92	27.4%	32	26.7%	80	28.9%	67	37.6%
Chance of dying over 10 years	231	37.7%	140	47.0%	120	35.7%	61	50.8%	111	40.1%	79	44.4%
Chance of needing a biopsy from screening over 10 years	73	11.9%	29	9.7%	43	12.8%	11	9.2%	30	10.8%	18	10.1%
Chance of impotence/incontinence over 10 years	137	22.3%	30	10.1%	81	24.1%	16	13.3%	56	20.2%	14	7.9%

In US men, the proportion choosing death as most important was lower (36%) for those choosing the no screening like-option compared with men who chose the screening-like option (51%), as might be expected; similarly, the proportion choosing incontinence/impotence as most important was higher (24% vs. 13%, respectively); and the proportion choosing chance of diagnosis or biopsy as most important did not differ based on unlabelled testing preference (overall chi^2^ with 3 df = 10.917, p = 0.012).

In Australian men, we observed a similar, but attenuated, pattern for the choice of mortality reduction, and a similar pattern for the choice of impotence or incontinence. Australian men who preferred the screening-like option were, however, more likely to choose chance of diagnosis as the most important attribute compared to those who preferred the no screening-like option. As in US men, there were no differences by testing preference in the proportion choosing chance of biopsy as most important (overall chi^2^ with 3df = 13.854, p = 0.003).

### Screening intent

Labelled screening intent was high amongst participants from both countries (mean intent score: Australia 4.04; US 3.95; p = 0.110). The proportion of participants who agreed or strongly agreed that they intended to have PSA testing when labelled as such was high and did not differ between groups (Australia, 78.6%; US 73.7% p = 0.130) (Table 4).

**Table 4:** Intent to be screened by country

	**Overall (n = 911)**		**US (n = 456)**		**AUS (n = 455)**		**p-value**
	**N**	**%**	**N**	**%**	**N**	**%**	
Strongly Disagree [1]	19	2.1%	12	2.6%	7	1.4%	0.441
Disagree	33	3.6%	20	4.4%	13	2.9%	
Neither Agree nor Disagree	168	18.4%	88	19.3%	80	17.6%	
Agree	399	43.8%	193	42.3%	206	45.3%	
Strongly Agree [5]	292	32.1%	143	31.4%	149	32.7%	
Mean Intent (SD)	4.00 (0.91)		3.95 (0.96)		4.04 (0.87)		0.110

## Discussion

We found that Australian and US men had similar preference structures with respect to attributes of PSA screening. When faced with an unlabelled question, about a third of men expressed a preference for the PSA-like option. Australian men were more likely to prefer the PSA-like option (over the no screening option) compared to US men. However, labelled intent to have PSA testing was high amongst both US and Australian men, with approximately three quarters of men indicating that they intended to be screened.

The finding that PSA screening was favoured by a greater proportion of Australian than US men on the unlabelled test preference question was unexpected: we had anticipated similar results between countries. Both countries have relatively high rates of screening on national surveys, [12,14] and this finding may have occurred by chance. However, it is also possible that the recent USPSTF guidelines [15], which were published in draft form in October 2011 (near the time of our data collection), may have had a (larger) effect on US men’s preferences. It is possible that doctors’ practices for discussing PSA testing may vary across countries, and that doctors in the US are less likely to discuss PSA testing than Australian doctors, although indirect evidence does not suggest this is the case [8,16].

Our findings have a number of implications. First, they suggest that the PSA label has a strong effect in each country: a large proportion of both US and Australian men intended to have PSA testing, despite their preference for the “no PSA” option in the unlabelled question. The observed labelling effect suggests that men may be unaware of the true characteristics of PSA testing or that there are other attributes of benefit of the test that are not captured in our study.

Indirect evidence suggests that men did not understand or appreciate that the effect of PSA on increasing the chance of prostate cancer diagnosis in and of itself should not always be considered a benefit of screening, because of overdiagnosis. Considering the chance of diagnosis to be the most important attribute was associated with choosing the PSA-like option in the unlabelled question, despite the fact that screening increases risk. The challenges in communicating the concept of overdiagnosis are considerable, and require increased attention in the future, both for this question and other screening issues [17].

Our study has some methodological limitations that must be considered. First, it was conducted among an on-line panel. Whether the effects we observed would differ in men making the screening decision in a clinical setting is unclear. We attempted to bolster the salience of the question by enrolling men of screening age and asked them to answer as if they were actually deciding about whether to be tested, but we did not measure actual screening behaviour. Future studies should do so. Given the online panel recruitment, our participants may not be completely representative of the population of US and Australian men in this age group; however they were broadly comparable on factors such as education, employment status and prior test experience. Whether under-represented populations would have different preferences is unknown. We did not present participants with a full decision aid and we did not assess knowledge specifically, making it difficult to sort out effects of understanding vs. those related to values and preferences. That said, our survey instrument contained sufficient information to frame the decision appropriately.

Our findings can be used to enhance the shared decision making process, with more attention given to ensuring that men understand the key features of the PSA test, and recognise the potential downside of an increased risk of diagnosis. Future studies should examine the effect of feeding back the results of values clarification methods, particularly when they stand in contrast to men’s stated preferences for whether or not to be tested. Discussion of values and test preference may help patients arrive at an informed, value-concordant decision.

## Conclusions

We found that Australian and US men had high intent for PSA screening but that fewer men in each country chose the PSA-like option on an unlabelled question. Australian men were slightly more enthusiastic for screening, even after adjustment for known confounders. Men in both countries did not clearly view increased risk of diagnosis without reduction in mortality (overdiagnosis) as an important negative aspect of screening, and more work needs to be done on how best to communicate that concept to men facing the PSA decision and other similar screening decisions.

## Competing interests

The authors declare that they have no competing interests.

## Authors’ contributions

All authors have made significant contributions to the published study. MP, KH, SH, AB were involved in the conception and design of the research study. KH, TC, MP were involved in data collection. KH, MP, AB carried out the analyses. All authors aided in interpretation of results and revision of the manuscript. All authors have read and approved the final manuscript.

## Pre-publication history

The pre-publication history for this paper can be accessed here:

http://www.biomedcentral.com/1472-6963/13/388/prepub
